# An Increase of Seawater Temperature Upregulates the Expression of *Vibrio parahaemolyticus* Virulence Factors Implicated in Adhesion and Biofilm Formation

**DOI:** 10.3389/fmicb.2022.840628

**Published:** 2022-03-08

**Authors:** Mélanie Billaud, François Seneca, Eric Tambutté, Dorota Czerucka

**Affiliations:** ^1^Biomedical Department, Scientific Center of Monaco, Monaco, Monaco; ^2^Marine Biology Department, Scientific Center of Monaco, Monaco, Monaco

**Keywords:** *Vibrio parahaemolyticus*, climate change, virulence factors, biofilm, marine pathogen ecology

## Abstract

Climate change driven seawater temperature (SWT) increases results in greater abundance and geographical expansion of marine pathogens, among which *Vibrio parahaemolyticus* (Vp) causes serious economic and health issues. In addition, plastic pollution in the ocean constitutes a vector for harmful pathogens dissemination. We investigate the effect of elevated SWT on the expression of genes implicated in adhesion and biofilm formation on abiotic surfaces in the clinical Vp strain RIMD2210633, which expresses hemolysins. Among the genes studied, the multivalent adhesion molecule-7 and the GlcNAc-binding protein A were involved in the adhesion of Vp to abiotic and biotic surfaces, whereas the type IV pili, the mannose-sensitive hemagglutinin, and the chitin-regulated pilins facilitate attachment and biofilm formation. Data presented here show that at 21°C, Vp is still viable but does not either proliferate or express the virulence factors studied. Interestingly, at 27°C and as early as 1 h of incubation, all factors are transiently expressed in free-living bacteria only and even more upregulated at 31°C. These results clearly show that increased SWT has an important impact on the adhesion properties of free-living Vp to plastic support and thus emphasize the role of climate change in the spread of this pathogenic bacteria.

## Introduction

*Vibrio parahaemolyticus* (Vp) is a marine bacterium including strains pathogenic to humans. It is mostly known to cause acute gastroenteritis from the consumption of contaminated and undercooked seafood (Daniels et al., 2000). In rare cases, septicemia and extraintestinal infections have been reported as a result of infected wounds from exposition to contaminated waters (Daniels et al., 2000).

*Vibrio* pathogenesis in humans is associated with the production of many virulence factors, among which thermostable direct hemolysin (TDH) and TDH-related hemolysin are considered to be the main pathogenic factors of Vp (Baker-Austin et al., 2010; Mahoney et al., 2010). TDH acts directly on red blood cells and has hemolytic, enterotoxin, and cytotoxic activities. Epidemiological studies show that most clinical isolates of Vp contain genes coding for TDH and TDH-related hemolysin, whereas only a few environmental isolates contain these genes (Theethakaew et al., 2013). However, recent studies identified TDH in 48% of environmental strains indicating that hemolysin plays a role in bacterial fitness in the environment (Gutierrez West et al., 2013).

There are two major life stages in *Vibrio* species: free-living or planktonic and surface-associated. On the one hand, attachment to abiotic surfaces may be a survival strategy that allows bacteria to survive in a nutrient-poor natural environment by providing a selective advantage through access to nutrients that accumulate at the liquid–surface interface and promotes biofilm formation (Dawson et al., 1981). On the other hand, adhesion to host tissues is the initial step necessary to infection by Vp (Navarre and Schneewind, 1999). Both events, attachment and biofilm formation of Vp, are mediated by the bacterium’s expression of functional type IV pili. The genome of Vp encodes two types of IV pilins: the mannose-sensitive hemagglutinin (MSHA) and the chitin-regulated (PilA) pilins (Makino et al., 2003). MSHA pilin facilitates adherence to abiotic and biotic (*e.g.*, chitin) surfaces, whereas PilA pilin enables cell aggregation and biofilm formation (Shime-Hattori et al., 2006). Vp is also capable of synthesizing another extracellular secreted protein that binds to chitin, the GlcNAc-binding protein A (GbpA; Makino et al., 2003). GbpA is involved in the attachment of Vp to chitin but also to human intestinal cells (Kirn et al., 2005; Zampini et al., 2005; Stauder et al., 2012). In addition, the multivalent adhesion molecule-7 (MAM-7) is a constitutively expressed surface protein that contributes to pathogen adhesion to host cells during the early stage of infection (Krachler et al., 2011). It binds two different proteins on the host’s cell surface: fibronectin and phosphatidic acid (Krachler and Orth, 2011).

The incidence of Vp disease outbreaks is increasing, and the geographic range of serious infection is expanding, driven in large by rising seawater temperature (SWT) (Martinez-Urtaza et al., 2010). The expansion of *Vibrio* species distribution, from the Southern tropics to more Northern waters, has been reported (Vezzulli et al., 2012; Baker-Austin et al., 2017; Froelich and Daines, 2020). However, little is known about the effects of seawater warming on the expression of virulence factors or the bacterial adhesion to different substrates (*i.e.*, biotic or abiotic), although both represent great implications in the dissemination of Vp worldwide.

Plastic waste representing approximately 80% of all marine litter is now ubiquitous on the surface of oceans, coastlines, and even in most remote parts of the ocean (Carney Almroth and Eggert, 2019). As all new surfaces are introduced to the marine environment, microplastic is rapidly colonized by bacteria (Harrisson et al., 2014). According to the literature, some potentially pathogenic species, including Vp, are able to colonize a wide range of plastics, independently of their chemical composition, *e.g.*, polyethylene, polypropylene, or polystyrene (PS) (Kirstein et al., 2016; Laverty et al., 2020). Potentially pathogenic Vp originating from an estuarine environment were discovered on microplastics in the mid-North Atlantic Ocean and in the North Baltic Sea (Zettler et al., 2013; Kirstein et al., 2016). These findings suggest that harmful microbes that colonize plastics in the marine environment may use microplastics to travel long distances and expand their geographic range across oceans (Rech et al., 2018). In addition, bacteria-contaminated microplastics are ingested by filter-feeders such as oysters and mussels, which are in turn consumed by humans. Although the transfer of pathogens from plastic-containing seafood to humans is still speculative (Barboza et al., 2018), there are great concerns about the role of microplastics in increasing the threats of seafood-borne illnesses. The main goal of this study was to investigate the effects of SWT increase on the expression of virulence factors implicated in adhesion and biofilm formation. The temperatures studied were 21°C, corresponding to the annual average temperature of the Mediterranean Sea, 27°C as the average temperature of tropical waters, and 31°C as an estimated average temperature in the near future by the Intergovernmental Panel on Climate Change (Intergovernmental Panel on Climate Change [IPCC], 2019). In this study, we used a culture-dependent approach in the laboratory to carry out gene expression quantification of virulence factors in seawater at different temperatures in free-living bacteria (*i.e.*, in suspension) and in bacteria adhering to the PS-bottom of Petri dishes.

## Materials and Methods

### Bacterial Strain and Growth Condition

The clinically isolated strain Vp RIMD2210633 serotype O3:K6 was provided by T. Honda from Osaka University Japan. To culture, Vp was routinely grown in a 5-ml tube at 37°C in Luria-Bertani LB medium containing 3% NaCl (standard condition).

### Bacterial Growth in Different Experimental Conditions

Overnight Vp culture in standard condition was centrifuged at 2,500 × *g* for 15 min at room temperature (RT). The pellet containing bacteria was suspended in 1 ml of 0.22-μm filtered seawater (FSW) to obtain a dilution corresponding to an optical density at a wavelength of 620 nm (OD_620_) ∼0.8. Sixty microliters of the bacterial solution was put into six-well plates with 2 ml of FSW and incubated at different temperatures, 21, 27, and 31°C. Bacterial growth was followed by measurement of OD_620_ until reaching the stationary phase.

### Bacterial Viability Test Determined by Fluorescence Intensity Measurement

For the viability measurement, overnight culture of bacteria was centrifuged at 2,500 × *g* for 15 min at RT. The pellets containing bacteria were suspended in 1 ml of FSW. Sixty microliters of the bacterial solution was put into six-well plates that were incubated at different temperatures, 21, 27, and 31°C, for 1 h. Samples were centrifuged at 2,500 × *g* for 15 min at RT. The pellet was suspended in a 0.85% NaCl Buffer. OD_620_ was adjusted to ∼0.06. The viability was measured using the LIVE/DEAD^®^ BacLight™ bacterial viability kit according to the manufacturer’s instructions using the Synergy H1 spectrophotometer. Bacterial samples were measured in triplicate. Each sample ratio obtained was compared with the standard curve. For the standard curve, alive Vps were conserved in 0.85% NaCl solution, and dead Vps were killed in 70% isopropyl alcohol solution. The standard curve was obtained by mixing Live/Dead bacteria in the following proportions: 0:100; 10:90; 50:50; 90:10; and 100:0.

### Scanning Electronic Microscopy

Vps were inoculated in 35-mm Petri dishes and maintained in 2 ml of seawater at different temperatures: 27 and 31°C. After 1 h incubation, the free-living bacteria were eliminated by successive washing with seawater, and bacteria adhering to the bottom of Petri dishes were fixed with 4% PFA/FSW (1v/2v). Samples were dehydrated by transfer through a graded series of ethanol, ending with a concentration of 100%. After dehydration, they were incubated for 30 min in hexamethyldisilazane (HMDS)/ethanol 100% (1v/2v), 30 min in HMDS/ethanol (v/v), and 2 × 30 min in HMDS 100% that was subsequently evaporated under a fume hood overnight. Samples were then coated with gold-palladium and observed at 3–5 kV with a JEOL JSM-6010LV.

### Measurement of the Amount of Biofilm

Vps were inoculated in a six-well plate dish in 1 ml of FSW. A volume of 180 μl was transferred into a 96-well plate. The plate with the bacterial suspension and the 96-ped Lid were incubated at different temperatures: 27 and 31°C for 1 h. Formation of biofilm was measured by crystal violet staining method using Biofilm Formation Assay Kit (Dojindo Laboratories) according to the manufacturer’s instructions.

### RNA Extraction From Free-Living Bacteria and Bacteria Attached to the Well Bottom

Overnight VPs grown in standard condition were centrifuged, and the pellet containing bacteria were suspended in 1 ml of FSW corresponding to OD_620_ ∼0.8. Sixty microliters of the bacterial solution was put into 1 ml of each medium per well of a six-well plate (Falcon- Ref 353046) and incubated at 21, 27, and 31°C. After 0.5, 1, 3, and 6 h, free-living bacteria were collected and pelleted by centrifugation at 4,500 × *g* for 15 min at RT. The supernatant is discarded. The pellet is suspended into 200 μl of Trizol max (Trizol Max Bacterial RNA Isolation Kit from Thermo Fisher Scientific).

For RNA extraction from bacteria attached to the plate bottom, 250 μl of Trizol max has been added directly into each well plate. The bacteria layer is scrapped, and approximately 200 μl is recovered. All samples are incubated at 95°C for 4 min. We added 1 ml of Trizol Reagent (Invitrogen) to each sample, mixed thoroughly, and waited 5 min at RT.

All the samples obtained from free-swimming and attached bacteria are grinding with Zirconium beads thanks to a Precellys Homogenizer at 2600 revolution two times for 30 s at RT. RNA was purified using columns from the DirectZol kit (Zymo Research) following the manufacturer’s instructions. Total RNA was quantified at 260-nm wavelength using a Synergy H1M spectrophotometer.

### Quantitative Reverse Transcription-Polymerase Chain Reaction

Bacterial RNA was used for the determination of the expression of virulence factors. Complementary DNA (cDNA) was constructed using the RevertAid First Strand cDNA Synthesis Kit (Fermentas/Thermoscientific, K1622) according to the manufacturer’s instructions.

The quantitative reverse transcription-polymerase chain reaction (RT-qPCR) was performed using the Applied Biosystems Real-Time PCR instrument. Fifteen nanograms of cDNA was placed in triplicate in each well of the qPCR plate, and a solution was added containing: 10 μM of forward primer, 10 μM of reverse primer, 0.1 μl of carboxy-X-rhodamine, which is a dye, 4.9 μl of RNAse free water, and 10 μl of SYBR, which is completed to obtain a final volume of 20 μl per well. The primers used are listed in Supplementary Table 1. The plate is then centrifuged. Efficiencies of the primers were in the range of 95–105%. Machine parameters are set to “Comparative Ct,” “SYBR Green Reagents” detected, including dissociation curves and on standard cycles (approximately 2 h). Quantification was determined using the comparative cycle threshold (C_T_) method relative to housekeeping gene RecA, which is highly conserved and implicated in DNA repair or genetic recombination. The average expression obtained after 30-min incubation of bacteria in different media was normalized to 1 for relative quantification expression (RQ).

### Statistical Analysis

The results are expressed as the mean ± standard error. Statistical studies were performed by analysis of means (*T*-test). The p-values < 0.05 are considered to be significantly different.

## Results

### Correlation Between *Vibrio parahaemolyticus* Growth and Viability Maintained in Different Seawater Temperatures

**FIGURE 1:**
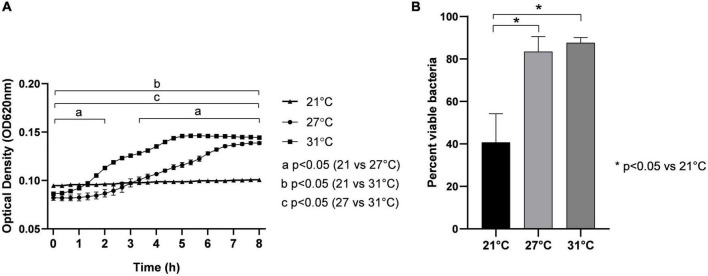
Effect of temperature on growth by optical density measurement at 620 nm (OD_620_) **(A)** and viability **(B)** of *V. parahaemolyticus* maintained in seawater at different temperatures: 21, 27, and 31°C. Bacteria were seeded in FSW at OD_620_ ∼0.1, corresponding to 3.4 × 10^7^ CFU/ml. Results are expressed as means ± standard deviations from experiments performed with three replicate samples.

We measured the growth and viability of Vp maintained at different SWT (21, 27, and 31°C). At 21°C, Vp did not grow (Figure 1A) over all the time of the experimentation (8 h), and after 1 h of exposure, only a small percentage of bacterium (around 40%) were viable (Figure 1B). By contrast, when maintained at 27°C, Vp grows and reaches a stationary phase after 7 h of incubation with OD_620_ ∼0.15. The bacterium growth was significantly faster at 31°C when compared with 27°C. It begins after 1 h of incubation at 31°C *versus* 3 h at 27°C. In both conditions (27 and 31°C), the estimated numbers of Vp (OD_620_ of 0.15) in the stationary phase were low (Figure 1A). These numbers represent 10% of the bacteria estimated in the stationary phase when maintained in LB NaCl 3% (Supplementary Figure 1). This indicates that seawater represents environmental conditions rather than the optimal medium to grow Vp. However, in both conditions (27 and 31°C), cell viability reaches values close to 90% (Figure 1B), similar to environmental conditions.

### Adhesion of *Vibrio parahaemolyticus* to the Bottom of Petri Dishes Is Influenced by Temperature

**FIGURE 2:**
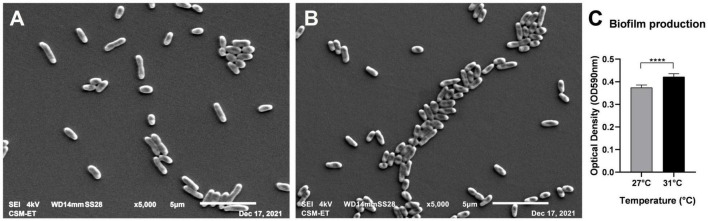
Scanning electron micrographs presenting adhering *V. parahaemolyticus* at 27°C **(A)** and 31°C **(B)**. (Magnification × 5,000). Biofilm quantification of adhering bacteria to plastic **(C)**. ****p-values < 0.0001.

Vps were inoculated in 35-mm Petri dishes and maintained in 2 ml of seawater at different temperatures: 27 and 31°C. Images illustrating adherent bacteria by scanning microscopy are presented in Figure 2A for 27°C and Figure 2B for 31°C. Bacterial clusters are larger in size at 31°C (Figure 2B). The effect of temperature on biofilm formation was confirmed by biofilm quantification (Figure 2C) significatively higher at 31°C (*n* = 8). This observation led us to investigate the effect of temperature on the expression of genes implicated in adhesion and biofilm formation.

### Increasing Seawater Temperature Upregulates Genes Expression Implicated in Adhesion and Biofilm Formation

**FIGURE 3:**
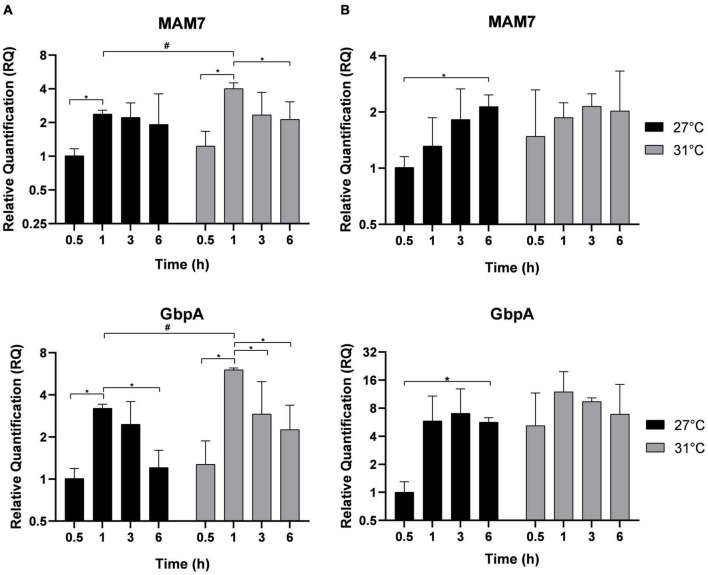
Adhesion genes expression MAM-7 and GbpA in free-living *V. parahaemolyticus* **(A)** and adherent bacteria **(B)** incubated at 27°C (black) and 31°C (gray). Bacteria were maintained 0.5, 1, 3, and 6 h in different conditions before RNA extraction from free-living bacteria and adherent bacteria for same well. Relative quantification expressions are expressed as means ± standard deviations of results from experiments performed with three replicate samples. ^#^Significant difference between temperatures. *Significant difference between time points.

Adhesion is the first step necessary for biofilm formation on abiotic surfaces or host infection. MAM-7 and GbpA play a role in the adhesion of Vp to abiotic and biotic surfaces (Zampini et al., 2005; Krachler et al., 2011; Stauder et al., 2012). After different times of incubation indicated in Figure 3, MAM-7 and GbpA genes expressions were estimated in free-living bacteria (Figure 3A) and in adherent bacteria (Figure 3B). In free-living bacteria, MAM-7 and GbpA gene expressions were transiently upregulated over kinetics with a maximal expression after 1 h at both temperatures. However, at this time point, the expression of both genes was significantly higher at 31°C compared with 27°C. After 1 h of incubation, the expression of both genes dropped significantly. In adherent bacteria (Figure 3B), we did not observe any significant modification in the expression of MAM-7 and GbpA at the early time of incubation, but we observed an overexpression of these genes in both conditions (27 and 31°C) after 6 h of incubation.

**FIGURE 4:**
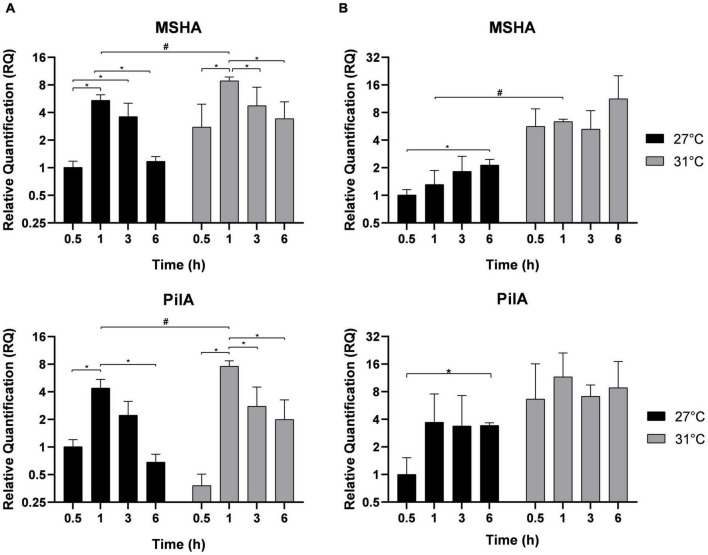
Biofilm genes expression MSHA and PilA in free-living *V. parahaemolyticus* **(A)** and adherent bacteria **(B)** incubated at 27°C (black) and 31°C (gray). Bacteria were maintained 0.5, 1, 3, and 6 h in different conditions before RNA extraction from free-living bacteria and adherent bacteria in same well. Relative quantification expressions are expressed as means ± standard deviations of results from experiments performed with three replicate samples. ^#^Significant difference between temperatures. *Significant difference between time points.

In Vp, the MSHA pili facilitate attachment to abiotic surfaces and biofilm formation, whereas PilA pili mediate cell-to-cell interactions and the binding of one bacterium to another (Shime-Hattori et al., 2006). After different incubation times, their expressions were estimated in free-living bacteria (Figure 4A) and in adherent bacteria (Figure 4B). The profile of their expression is similar to those found for MAM-7 and GbpA. In free-living bacteria, MSHA and PilA expressions were overexpressed after 1 h of incubation when compared with the first time point 0.5 h, and their expressions were significantly higher at 31°C compared with 27°C. After 3 and 6 h of incubation, the expression of both genes significantly dropped. In adherent bacteria (Figure 4B), we did not observe a significant modification in the expression of MSHA and PilA between the two temperatures 27 and 31°C in the early time of incubation, but we observed overexpression in both conditions at 6 h of incubation.

Note that the expression of all these genes is not upregulated at 21°C in free-living bacteria (Supplementary Figure 2) nor in adherent bacteria (Supplementary Figure 3).

### Increasing Seawater Temperature Induces the Expression of Thermostable Direct Hemolysin

**FIGURE 5:**
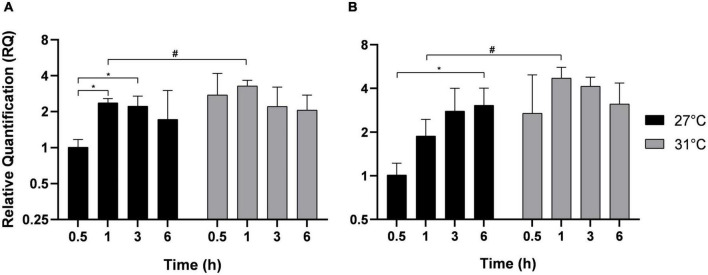
Thermostable direct hemolysin gene expression in free-living *V. parahaemolyticus* **(A)** and adherent bacteria **(B)** incubated at 27°C (black) and 31°C (gray). Bacteria were maintained 0.5, 1, 3, and 6 h in different conditions before RNA extraction from free-living bacteria and adherent bacteria in same well. Relative quantification expressions are expressed as means ± standard deviations of results from experiments performed with three replicate samples. ^#^Significant difference between temperatures. *Significant difference between time points.

Thermostable direct hemolysin is a hemolysin involved in the pathogenesis of Vp strains that infect humans. As the strain 03:K6 was isolated from a patient, we looked at whether these bacteria synthesize TDH in seawater and whether the increase in temperature can influence its expression level. After different incubation times, TDH gene expression was estimated in free-living bacteria (Figure 5A) and in adherent bacteria (Figure 5B). In free-living bacteria, a significant increase in TDH gene expression was observed at 27°C after 1 and 3 h of incubation compared with the first time point at 0.5 h. In comparison, no significant increase in TDH expression was observed over time at 31°C. Similarly, the TDH transcript level was not modified at 21°C in both bacterial populations (Supplementary Figure 4). However, after 1 h of incubation, TDH expression was significantly upregulated at 31°C compared with 27°C (Figures 5A,B) in both free-living and adherent bacteria.

## Discussion

Bacterial adhesion is essential to the colonization of biotic and abiotic surfaces and represents a link between the behavior of bacteria in natural environments and their pathogenicity potential. Data presented in this study show that elevated SWTs can affect the expression of genes involved in adhesion and biofilm formation in Vp and favor its adhesion to plastics. Warmer temperatures also promote Vp’s pathogenicity by inducing the synthesis of the TDH toxin. Interestingly, bacterial cultures in the laboratory consisting of suspended and adherent populations (*i.e.*, similar to free-living planktonic and sedentary in the environment) show differences in the kinetics and induction rates of all these genes.

Pathogenic bacteria must detect and respond to changes in their local environment parameters (*i.e.*, temperature, pH, salinity, and nutrient availability) to successfully colonize and invade their host (Gode-Potratz et al., 2011). Among pathogens, vibrios show a broad tolerance to several environmental parameters, including temperature. In this study, SWT significantly influences the growth of the clinical Vp strain O3:K6, which is latent at 21°C but begins to grow at 27 and 31°C. This result confirms previous observations that under adverse conditions, including cold temperature, Vp is able to enter a viable but not culturable state that supports long-term survival (Xu et al., 1982; Oliver, 2005; Burnham et al., 2009). Entering a partial dormant state is one of the ways Vp can persist in cold environments. Attachment to substrates such as plankton has also been proposed as another mechanism of environmental persistence (Sarkar et al., 1983).

Because adhesion and biofilm formation is the first step in the colonization of surfaces and pathogenesis, we studied the influence of temperature on the expression of genes essential to these mechanisms: adhesins and pilins. Our data show a quick induction of the expression of genes in free-living bacteria coding for adhesins (MAM-7 and GbpA) and the type IV pilins (MSHA and PilA) occurring after 1 h of incubation at 27°C. Warmer temperature exerts a stimulating effect, as these genes are upregulated at 31°C when compared with 27°C. After 3 and 6 h of incubation, expression levels of GbpA, PilA, and MSHA are downregulated. Interestingly, the quick increase in gene expression at 1 h is observed only in suspended bacteria. In adherent bacteria, all the genes mentioned earlier are upregulated after 6 h of incubation, but the increased temperature has no impact on their expressions. Our data show that an adaptation of Vp to temperature fluctuations results in the regulation of adhesin and pilin genes. However, this temperature-dependent regulation only occurs in suspended and not in adherent bacteria.

Differences in gene expression between free-living and sedentary lifestyles have been described for many pathogenic bacteria and involve surface sensing (Gode-Potratz et al., 2011). In the case of flagellated rod-shaped bacteria (*e.g.*, *E. coli* and vibrios), surface sensing takes place *via* a two-step process: (1) reversible attachment characterized by polarly attached bacteria and (2) irreversible attachment, which results in microcolonies and biofilm formation (Armbruster and Parsek, 2018). Vp senses the surface *via* rotation of the flagella and can stimulate a specific program of genes depending on whether the surface is favorable to colonization and pathogenesis. The OpaR gene has been described as a quorum-sensing regulator that negatively regulates polar flagellum in Vp (Lu et al., 2021). Here, OpaR is upregulated in suspended Vp after 1 h of incubation (Supplementary Figure 5A) and suggests repression of Vp’s motility by regulating the expression of its flagella. Noteworthy, this gene is also overexpressed at 31°C compared with 27°C, further supporting our previous observation on the increased adhesion at elevated temperature.

The two pilin genes studied play different roles in the adhesion process. MSHA pilin is crucial to arresting cell motion in near-surface swimming bacteria and allows the transition to irreversible attachment and colony formation, whereas PilA pilin mediates cell-to-cell interactions and binding of one bacterium to another (Frischkorn et al., 2013; Utada et al., 2014). Both pilins, MSHA and PilA, worked synergistically and were involved in biofilm formation in Vp on abiotic PS, synthetic chitin, and biological diatom-derived chitin (Frischkorn et al., 2013). Data presented here clearly show that these two pilin genes are synergistically expressed in suspended Vp. We also observed a significant increase in expression for these two genes at 31°C, which could potentially also promote the attachment and the formation of colonies.

The role of adhesins in colony formation on plastics is less studied. GbpA and MAM-7 were mainly implicated in the virulence processes. GbpA contributes to the adhesion on culture epithelial cells by interacting with GlcNAC residues of mucin and interacting with natural polymers, *e.g*., chitin present on marine hosts (Zampini et al., 2005; Bhowmick et al., 2008). MAM-7 constitutes an adhesin especially found in mammal infection cases (Krachler et al., 2011). This adhesin binds with high affinity to phosphatidic acid and low affinity to fibronectin (Krachler and Orth, 2011). To our knowledge, there are no reports on the binding of GbpA and MAM-7 to synthetic polymers. Curiously, this adhesin is upregulated in seawater in free-living bacteria only, and its expression is positively influenced by elevated temperature. The role of GbpA and MAM-7 in biofilm formation on synthetic polymers, *e.g.*, PS of the bottom of Petri dishes, requires further investigation.

As the body temperature of mammals is often higher than the temperature of their external environment, many bacterial virulence factors are thermally regulated. TDH is a virulence factor mainly present in clinical isolates of Vp. The Vp strain O3:K6 used in this study is isolated from a patient, and this strain was reported to synthesize TDH at 37°C (Mahoney et al., 2010). Data presented here show that this strain expresses TDH in seawater at higher levels at 31°C when compared with 27°C. These data first illustrate that this clinical strain can express TDH in seawater and, second, that its expression is temperature-dependent. Thermoregulation of the expression of known and putative virulence-associated traits, including hemolysin, motility, and biofilm formation, has only been reported in environmental and clinical strains maintained in a heart infusion medium (Mahoney et al., 2010). To our knowledge, this is the first study to demonstrate the effect of temperature on the upregulation of TDH expression in a clinical Vp strain maintained in seawater. It is also interesting to point out that this toxin is synthesized in free-living and adherent bacteria.

A direct link between infection of marine animals and temperature-dependent expression of virulence factors was recently reported in the case of *Vibrio coralliilyticus* that was isolated from the coral *Acropora cytherea* during a bleaching outbreak due to thermal stress (Ushijima et al., 2016). In the case of this vibrio, mutation of MSHA reduces its virulence. Another mutation in ToxR also decreases the pathogenicity of this bacterium. The ToxR gene encodes a regulatory protein under the control of quorum-sensing and is involved in regulating the expression of virulence genes in vibrios (Zhang et al., 2018). Here, ToxR is overexpressed in suspended Vp during the early time of incubation and is upregulated at 31°C when compared with 27°C (Supplementary Figure 5B). These results support the correlation between SWT and Vp’s virulence.

As plastic debris in the environment continues to increase, an emerging concern is the potential for microplastics to act as vectors in pathogen dissemination. Zettler et al. (2013) showed that microbial communities on marine plastic debris differ consistently from the surrounding seawater communities and coined the term “Plastisphere” for this new habitat, which is now acting as a “mobile ecological niche.” Numerous reports describe the presence of pathogenic bacteria on both macro- and microplastic surfaces found across oceans (Amaral-Zettler et al., 2020). Among marine bacteria, *Vibrio* spp. have been found in high abundances within the “plastisphere,” in particular during summer months (Bowley et al., 2021). This observation is in agreement with the findings reported in this study, showing that elevated SWTs favor the expression of genes implicated in the adhesion of *Vibrio* to plastic and, consequently, the number of adhering bacteria. In addition, several studies demonstrate that microplastic surfaces represent favorable environments for biofilm formation. Living within a biofilm is highly beneficial for adhering bacteria to become more infectious than free-living bacteria. When compared with free-living bacteria, adhering bacteria have shown significant elevation in metabolic pathways that contributes to pathogenesis and favor horizontal gene transfer that may contribute to the development of resistance to antibiotics (Bowley et al., 2021). These observations also support our results showing different gene expressions for adhesins between free-living and adhering bacteria.

Altogether, the findings presented in this study clearly show the impact of increased temperature on the virulence of Vp in seawater environments. Warmer temperatures promote growth and the expression of virulence factors implicated in biofilm formation on abiotic surfaces and the synthesis of toxins. These factors play an important role in the pathogenesis of Vp and therefore represent a mechanism by which increased SWT and plastic pollution can promote the spread of plastic-associated pathogenic Vp and the intensification of their virulence.

## Data Availability Statement

The original contributions presented in the study are included in the article/Supplementary Material, further inquiries can be directed to the corresponding author/s.

## Author Contributions

DC conceived and designed the experiments. MB and ET performed the experimentation. DC, MB, and FS analyzed the data. DC wrote the first draft. FS and MB revised the manuscript. All authors contributed to the article and approved the submitted version.

## Conflict of Interest

The authors declare that the research was conducted in the absence of any commercial or financial relationships that could be construed as a potential conflict of interest.

## Publisher’s Note

All claims expressed in this article are solely those of the authors and do not necessarily represent those of their affiliated organizations, or those of the publisher, the editors and the reviewers. Any product that may be evaluated in this article, or claim that may be made by its manufacturer, is not guaranteed or endorsed by the publisher.

## Supplementary Material

Supplementary Figure 1 Growth of Vp in LB NaCl 3%. Overnight *V. parahaemolyticus* culture in standard condition was centrifuged at 2500 x g during 15 min at room temperature (RT). The pellet containing bacteria was suspended in 1 mL of LB NaCl 3% to obtain a dilution corresponding to OD_620_ ∼ 0.8. 60 μl of the bacterial solution was put into 6 well plates with 2 mL of LB NaCl 3% and incubated at different temperatures 21, 27, 31, and 37°C. Bacterial growth was followed by measurement of OD_620_ until reaching the stationary phase.

Supplementary Figure 2 Adhesion and biofilm genes expression at 21°C in free-living Vp maintained in FSW.

Supplementary Figure 3 Adhesion and biofilm genes expression at 21°C in adhering Vp maintained in FSW.

Supplementary Figure 4 TDH expression in free-living **(A)** or adhering **(B)** Vp maintained in FSW at 21°C.

Supplementary Figure 5 OpaR **(A)** and Tox R **(B)** expression in free-living Vp maintained in FSW at 27 and 31°C.

Supplementary Table 1 Primers and sequences used in this study.
