# Supplementary figures and images for: An Increase of Seawater Temperature Upregulates the Expression of Vibrio parahaemolyticus Virulence Factors Implicated in Adhesion and Biofilm Formation
Source: Front Microbiol. 2022 Mar 8;13:840628. doi: 10.3389/fmicb.2022.840628 (PMC8957992; doi:10.3389/fmicb.2022.840628)

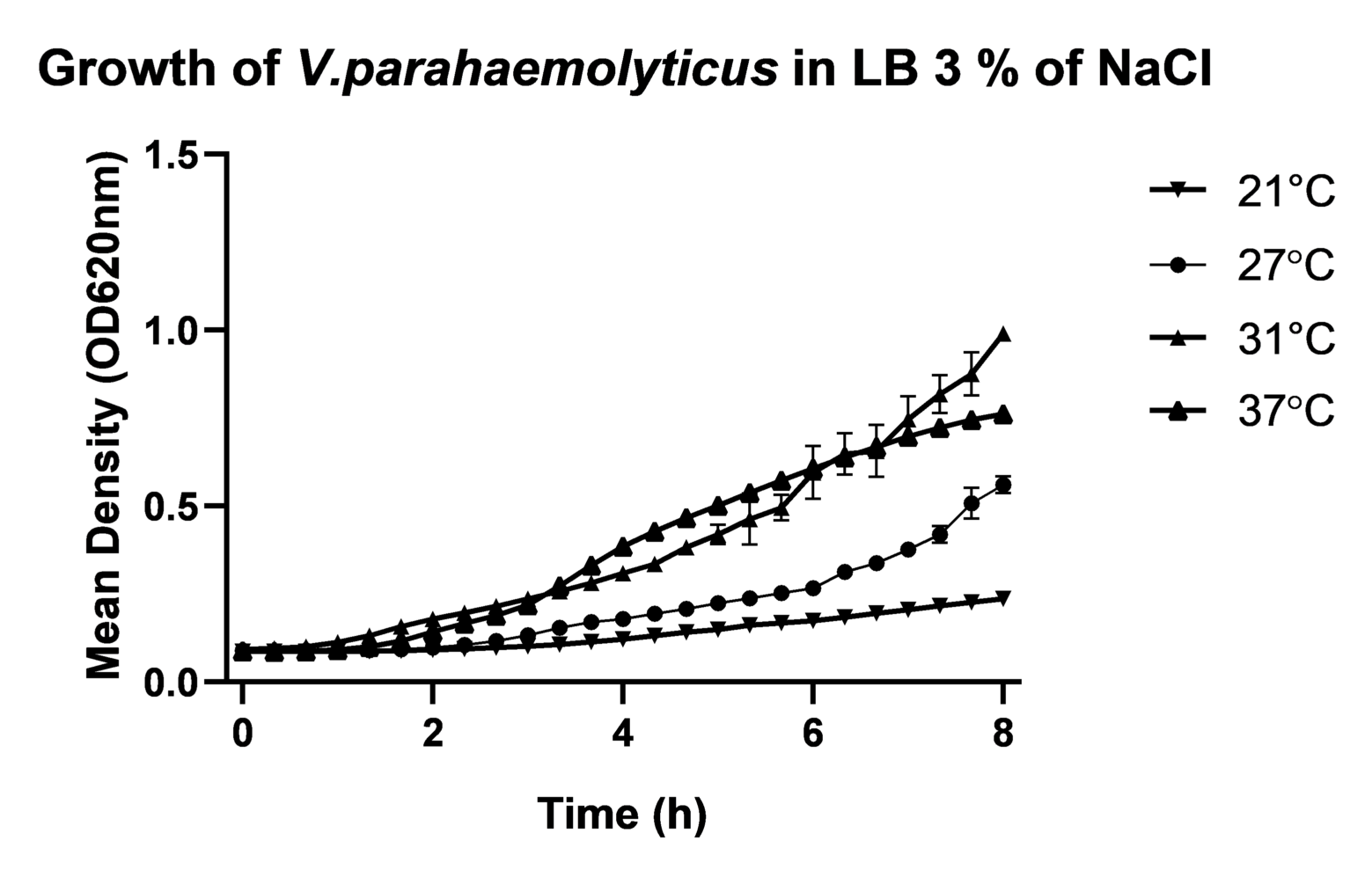

Supplement: Supplementary Figure 1 — Growth of Vp in LB NaCl 3%. Overnight V. parahaemolyticus culture in standard condition was centrifuged at 2500 x g during 15 min at room temperature (RT). The pellet containing bacteria was suspended in 1 mL of LB NaCl 3% to obtain a dilution corresponding to OD620 ∼ 0.8. 60 μl of the bacterial solution was put into 6 well plates with 2 mL of LB NaCl 3% and incubated at different temperatures 21, 27, 31, and 37°C. Bacterial growth was followed by measurement of OD620 until reaching the stationary phase. [file Image_1.TIF]

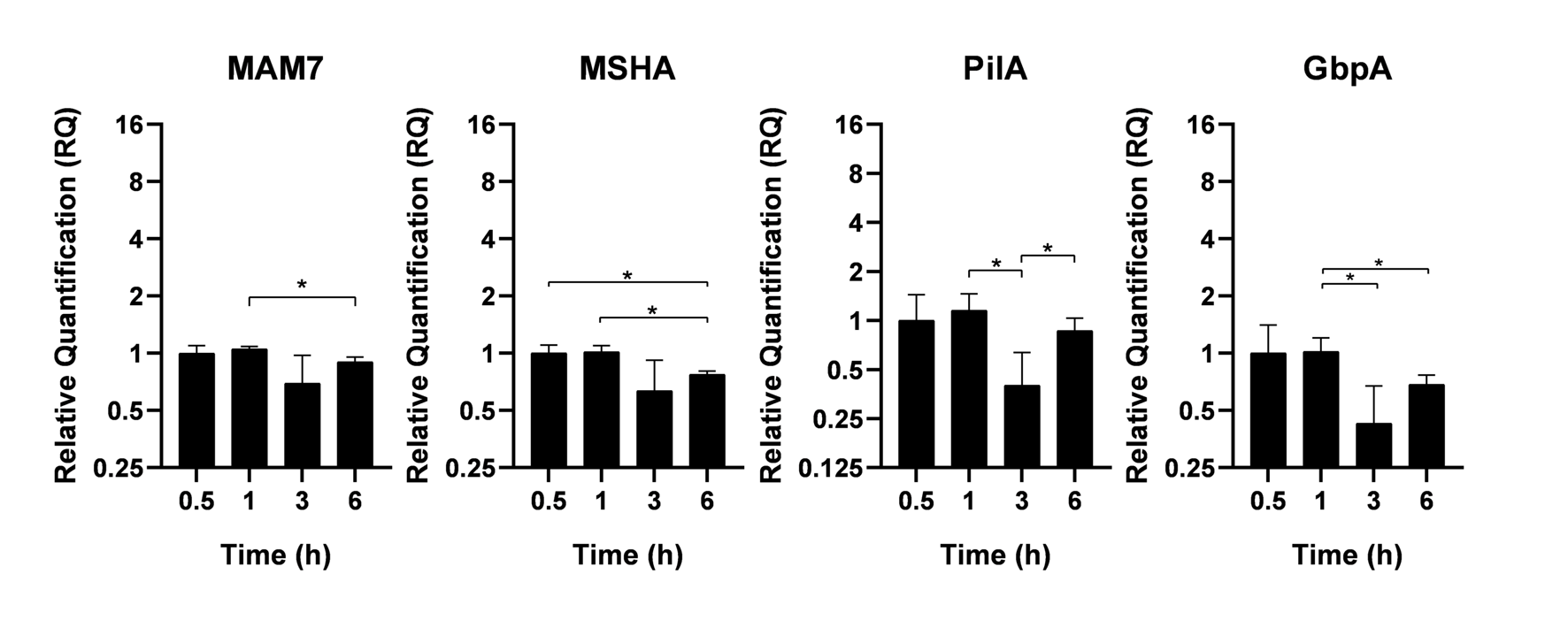

Supplement: Supplementary Figure 2 — Adhesion and biofilm genes expression at 21°C in free-living Vp maintained in FSW. [file Image_2.TIF]

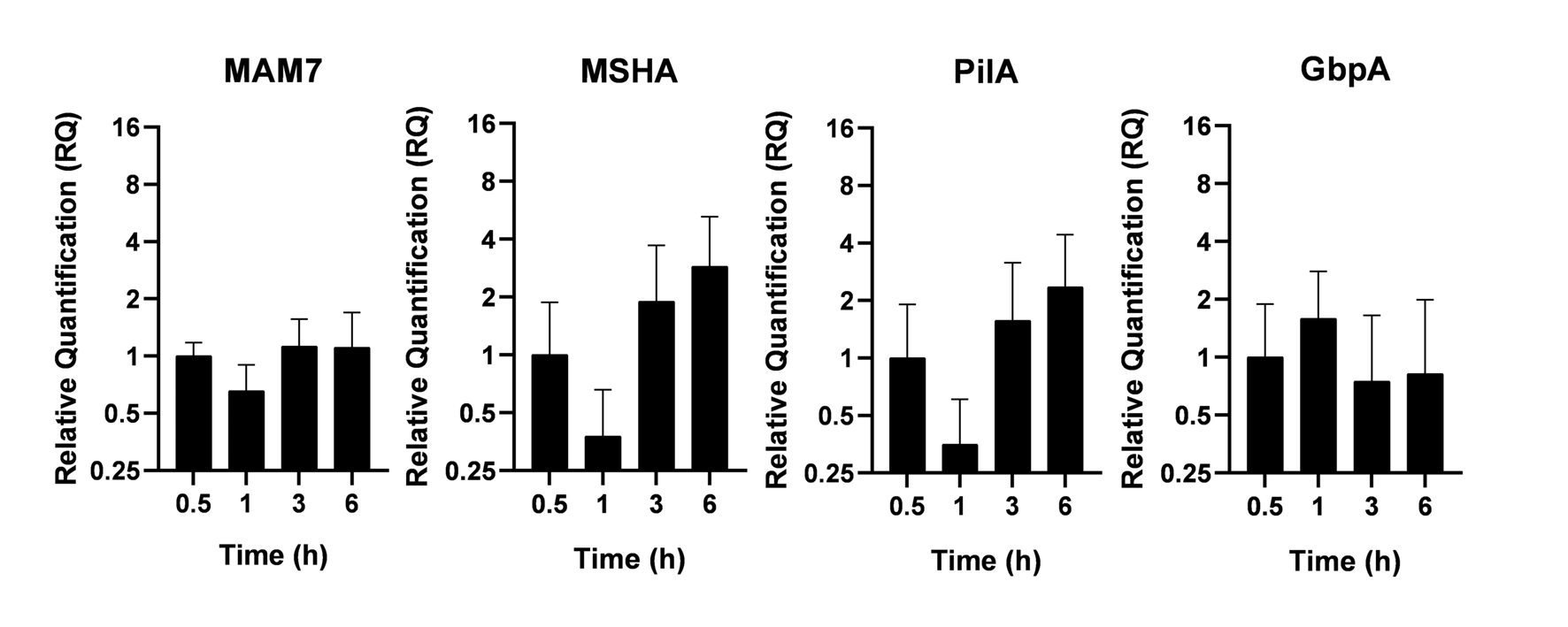

Supplement: Supplementary Figure 3 — Adhesion and biofilm genes expression at 21°C in adhering Vp maintained in FSW. [file Image_3.TIF]

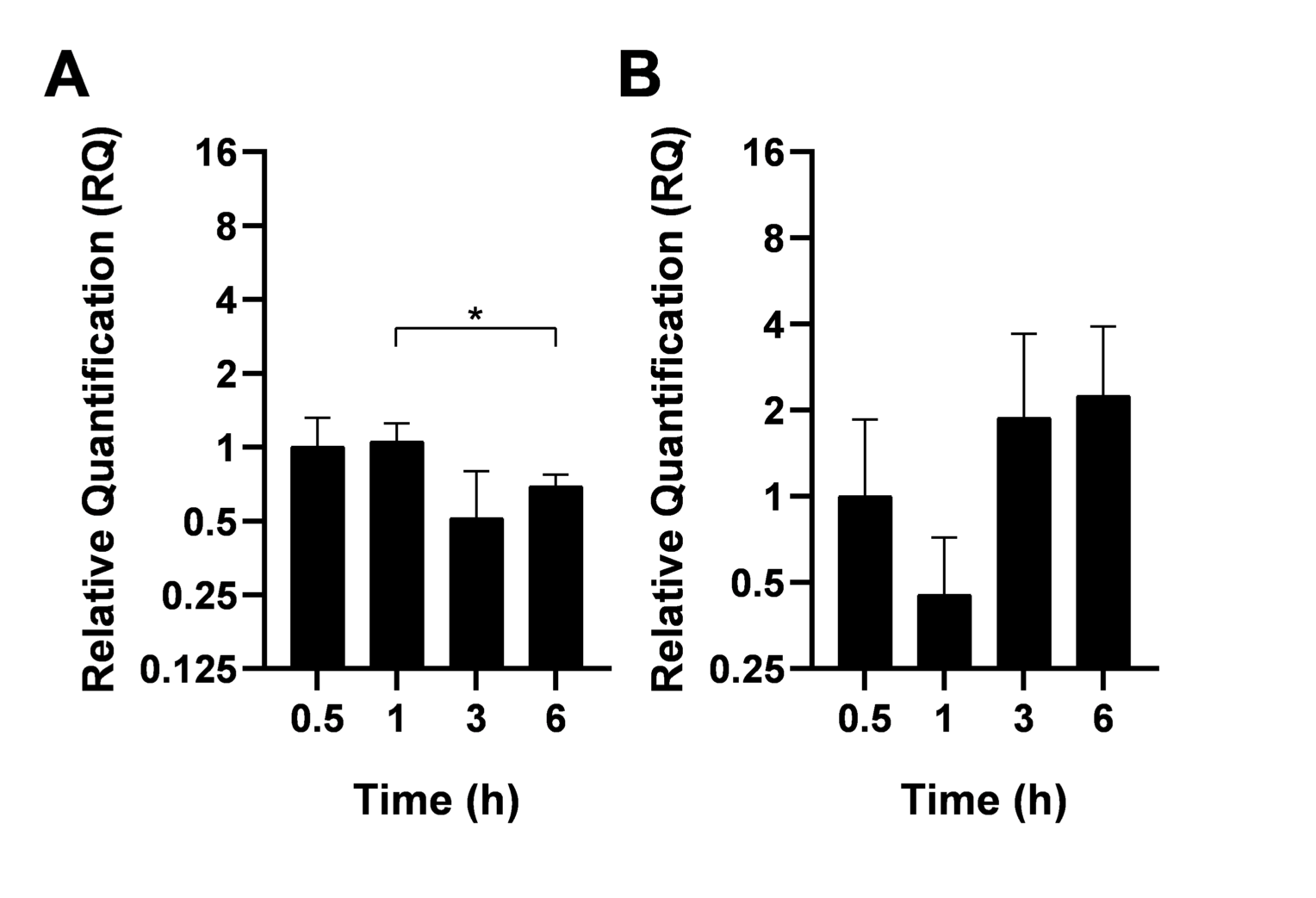

Supplement: Supplementary Figure 4 — TDH expression in free-living (A) or adhering (B) Vp maintained in FSW at 21°C. [file Image_4.TIF]

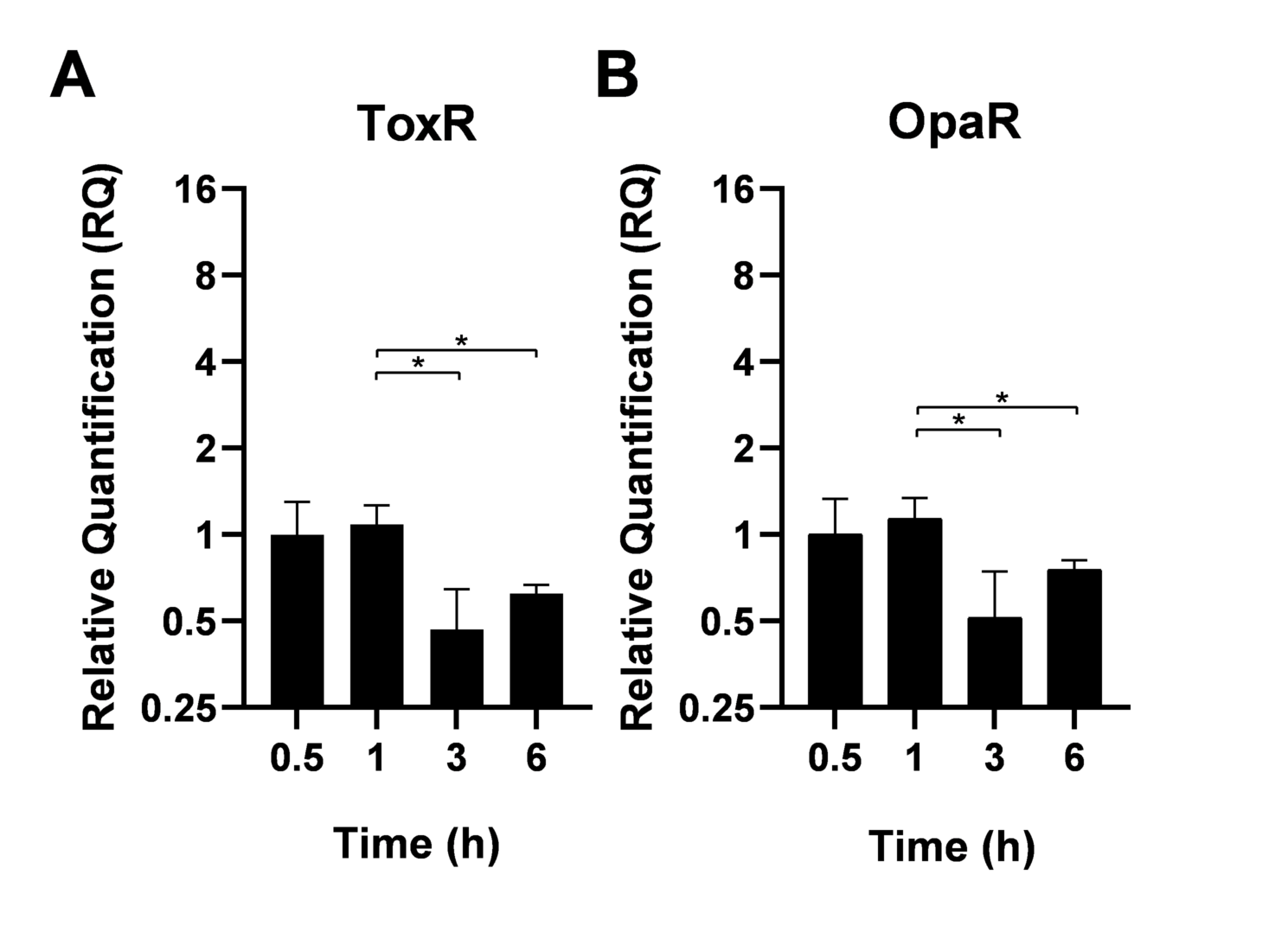

Supplement: Supplementary Figure 5 — OpaR (A) and Tox R (B) expression in free-living Vp maintained in FSW at 27 and 31°C. [file Image_5.TIF]
