# Supplementary material for: An Increase of Seawater Temperature Upregulates the Expression of Vibrio parahaemolyticus Virulence Factors Implicated in Adhesion and Biofilm Formation
Source: Front Microbiol. 2022 Mar 8;13:840628. doi: 10.3389/fmicb.2022.840628 (PMC8957992; doi:10.3389/fmicb.2022.840628)
Supplement: Supplementary Table 1 — Primers and sequences used in this study. [file Table_1.DOCX]

| Table 1 | Primers and sequences | |  |
| --- | --- | --- | --- |
|  |  |  |  |
| Target Gene | Direction | Primer sequence (5'-3’) | Reference |
| RecA | Forward | GCTAGTAGAAAAAGCGGGTG | (Ma YJ, Sun XH, Xu XY, et al. 2015) |
|  | Reverse | GCAGGTGCTTCTGGTTGAG |  |
| TDH | Forward | ATCTGTCCCTTTTCCTGCCC | This study |
|  | Reverse | ACGGTTTGTCCAAAAGTCAGAG |  |
| MAM7 | Forward | TTTGGCGTCAGCAGCAATTC | This study |
|  | Reverse | CGCCAGGAACGATAGTGAGG |  |
| GbpA | Forward | CCTCACTCTTACCGCAGAGG | This study |
|  | Reverse | CAAGGTTGCCATCGGTATCT |  |
| MSHA | Forward | GCTTAGCCGTACTCGTTGCT | This study |
|  | Reverse | TGTAGTTGGCGGAGCATACA |  |
| PilA | Forward | GGCTGAGCTGCATTACCAAG | This study |
|  | Reverse | TTGCAGCGTCTCTTGTGAAC |  |
| ToxR | Forward | GAACTGAGATTCCGCTGGGT | This study |
|  | Reverse | GAACCAGAAGCGCCAGTAGT |  |
| OpaR | Forward | TGTCTACCAACCGCACTAACC | This study |
|  | Reverse | TTGAAGCGGTTTGCTTGTACG |  |

Ma YJ, Sun XH, Xu XY, et al. Investigation of Reference Genes in Vibrio parahaemolyticus for Gene Expression Analysis Using Quantitative RT-PCR. *PLoS One*. 2015;10(12):e0144362. Published 2015 Dec 11. doi:10.1371/journal.pone.0144362
